# Weighing up the options: childhood intervention to tackle obesity

**DOI:** 10.1038/s41390-025-04483-2

**Published:** 2025-10-09

**Authors:** Owen R. Vaughan, Dino A. Giussani

**Affiliations:** 1 Department of Maternal and Fetal Medicine, EGA Institute for Women’s Health, University College London, London, UK; 2 Department of Physiology, Development and Neuroscience, University of Cambridge, Cambridge, UK; 3 Loke Centre for Trophoblast Research, University of Cambridge, Cambridge, UK; 4 Strategic Research Initiative in Reproduction, University of Cambridge, Cambridge, UK; 5 Cardiovascular Strategic Research Initiative, University of Cambridge, Cambridge, UK

Obesity is rising in prevalence throughout the population, including in children. In the UK, one in seven children aged 2–15 lives with obesity. Obesity can begin very early, with 12% of toddlers and primary school-aged children affected. Childhood obesity is associated with higher blood pressure and increased cardiovascular risk in adulthood.^1^ Since cardiovascular disease causes a quarter of all deaths and costs the economy £29 billion per year in the UK, childhood obesity has a significant impact on the population’s health, which is of growing concern. Glucagon-like peptide-1 (GLP-1) receptor agonists like liraglutide and semaglutide are effective treatments for weight loss in adults with obesity and have several cardiovascular benefits. In this issue of *Pediatric Research*, Romariz et al.^2^ show that GLP-1 agonists are also effective for treating obesity in children, in a meta-analysis of randomised controlled trials.

Romariz and colleagues show that GLP-1 agonists decrease body weight, body mass index (BMI), and waist circumference in children aged 6–19 with obesity. Notably, they show that GLP-1 agonists reduce BMI specifically in children aged less than 12 years. The authors used a meta-analysis of 11 randomised controlled trials incorporating 1024 patients. The findings are important because they identify GLP-1 agonists as an effective pharmacological intervention to alleviate the symptoms of obesity in children, thereby improving their quality of life. Before this study, smaller meta-analyses had shown beneficial weight loss effects of GLP-1 agonists in patients under 18 but had not specifically determined their efficacy in children under 12. There are currently limited treatment options for children with obesity in this younger age group. The European Medicines Agency recently recommended authorising liraglutide for weight loss in children under 12, following publication of a year-long randomised trial in 82 children showing a beneficial reduction in BMI.^3^ The study by Romariz et al. provides further evidence supporting the use of GLP-1 agonists as a weight management tool in primary school-aged children. In addition, the study demonstrates that GLP-1 agonists are effective for weight loss irrespective of whether the patient has type 2 diabetes, and of the type of agonist used. This research is important because it has the potential to positively impact the health of the entire population and lower healthcare costs dramatically, as treating obesity in children will mitigate cardiovascular risk factors in later life.

Whilst the synthesis of Romariz and colleagues supports the proposal that GLP-1 agonists are effective for weight loss in patients younger than 12, this is based only on 2 randomised controlled trials, both of which used liraglutide. Only one of the trials included in the systematic review used the next-generation GLP-1 agonist semaglutide to treat obesity in adolescents aged 12–18 years, and none used the combined GLP-1 and glucose-dependent insulinotropic peptide agonist, tirzepatide. Both semaglutide and tirzepatide induce greater weight loss and are longer lasting than liraglutide, so they require less frequent injection. The authors also establish that GLP-1 agonists increase the risk of adverse gastrointestinal events, including diarrhoea and nausea in children, in line with their known side effects in adults. Promisingly, the risk conferred is smaller in younger children, and few patients discontinue treatment because of serious adverse effects, supporting the safety of these medications. Nevertheless, there is scope for further investigation in the youngest children with obesity to optimise weight loss treatment regimens and minimise side effects, important factors to ensure adherence to medication.

Outside of pharmacotherapy, current options for managing obesity in children are either lifestyle interventions or bariatric surgery. A recently updated series of Cochrane systematic reviews concluded that diet and activity lifestyle interventions have, at best, modest and short-lived beneficial effects in treating childhood obesity, and the relative efficacy of diet, activity or combined interventions depends on age.^4^ By contrast, a meta-analysis of bariatric surgery studies found that surgical intervention produces sustained weight loss and has large positive effects both on quality-of-life scores and cardiovascular risk factors in patients under 18 with obesity.^5^ However, bariatric surgery has complications and is challenging to scale up to cater to larger patient numbers. Pharmacological weight management with GLP-1 agonists is consequently an attractive option for managing obesity in children.

Even though GLP-1 agonists are safe and effective in children, there may be practical, economic and ethical challenges to pharmacological weight management in childhood. Obesity disproportionately affects children living in areas of deprivation and from ethnic minority groups. Therefore, equitable access to medication and inclusive guidelines are required to avoid magnifying these disparities. The GLP-1 agonists currently available require daily or weekly subcutaneous injection. Weight regain occurs rapidly within months of cessation of treatment, so patients are likely to require chronic medication. Given the high prevalence of obesity, this will incur a substantial economic cost to be borne either by the patient or the state. Remedying the fundamental determinants of the obesogenic environment that children are exposed to would be preferable. This could entail education and regulation influencing physical inactivity and the availability and overconsumption of unhealthy food and sugary drinks, and their social and commercial determinants. Certainly, increased taxation of sugary drinks has beneficially reduced intake in several countries.^6^ The biomedical community needs to provide clear evidence to healthcare regulators and policymakers to weigh up the relative benefits and costs of interventions aiming to mitigate obesity in children.

**Fig. 1:**
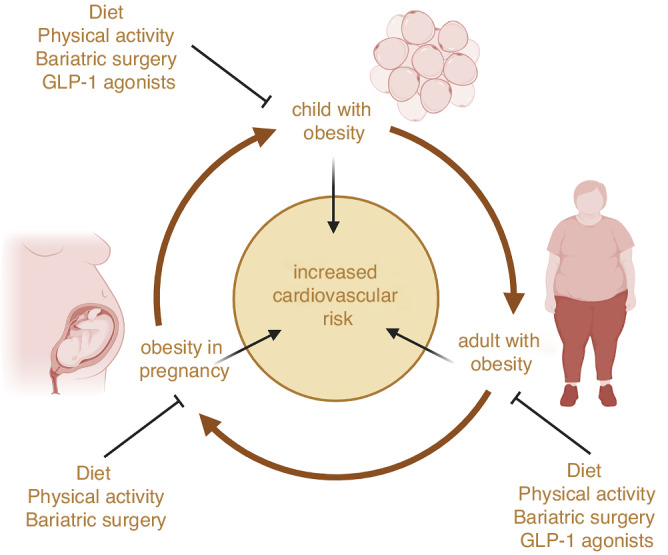
Opportunities for early intervention to reduce childhood obesity and prevent the intergenerational propagation of an increased cardiovascular risk. Created with BioRender.

There are also epigenetic determinants to consider, since children are more likely to have obesity if their mother had a high BMI before conception. More than half of women of reproductive age enter pregnancy overweight or living with obesity. Obesity in pregnant women also increases cardiovascular dysfunction in the adult offspring, as well as the mother.^7^ In women with obesity, pre-pregnancy weight loss via bariatric surgery reduces the rate of obesity in their children and improves their insulin sensitivity, lipid profile and markers of cardiometabolic risk, compared to children born before maternal bariatric surgery.^8^ Lifestyle interventions are also effective in reducing the incidence of pregnancy complications in pregnant women with obesity, but tend to induce only modest improvements in offspring cardiovascular outcomes without reducing adiposity. Lifestyle changes to optimise preconception health may be more effective. In preclinical studies, exercise and pharmacological interventions in animal models of maternal obesity improve cardiometabolic health in the offspring.^9,10^ For example, normalising maternal circulating adiponectin concentrations in a mouse model of maternal obesity mitigates excess fat accumulation and prevents cardiac dysfunction in the adult offspring.^9^ GLP-1 agonists are contraindicated during pregnancy due to safety concerns, because they restrict foetal growth when given to experimental animals. However, there is little evidence that they cause congenital abnormalities in the fetuses of women taking them inadvertently around conception.^11^ There is a distinct scarcity of information on their effects and the mechanism of action during pregnancy. Clearly, more research is needed to address this gap in knowledge on the role of maternal periconceptional and gestational interventions in managing childhood obesity, particularly in humans. However, Romariz et al. provide compelling evidence that GLP-1 agonists are a safe and effective option in primary school-age children, bringing preventative medicine forward and taking a step closer to tackling obesity and improving population health. Intervening early in children to tackle obesity will also improve the health of prospective parents and lower the cardiovascular risk in mothers and their offspring, breaking the intergenerational cycle of cardiometabolic disease risk propagation (Fig. 1).
